# Transcriptome analysis of 3D4/21 cells expressing CSFV NS4B

**DOI:** 10.3389/fmicb.2025.1510058

**Published:** 2025-02-04

**Authors:** Wang Dong, Huifang Lv, Yuzhen Song, Yujin Lv, Xiapeng Xu, Huiyuan Jing, Zhifeng Peng, Xinghui Song, Yongbin Guo

**Affiliations:** ^1^Key Laboratory of Veterinary Biological Products, College of Veterinary Medicine and Pharmacy, Henan University of Animal Husbandry and Economy, Zhengzhou, China; ^2^Agriculture and Rural Affairs Bureau, Dingzhou, China

**Keywords:** RNA-seq, classical swine fever virus, NS4B, endocytosis, autophagy

## Abstract

Classical swine fever (CSF) caused by classical swine fever virus (CSFV) has resulted in severe losses to the pig industry worldwide. CSFV non-structural protein 4B (NS4B) plays a crucial role in CSFV replication and pathogenicity. However, the function of NS4B is still limited during CSFV infection. In this study, the RNA-seq was used to investigate differentially expressed genes (DEGs) in 3D4/21 cells expressing CSFV NS4B. 4397 DEGs were identified in 3D4/21 cells expressing NS4B compared to cells expressing the empty vector (NC). Twelve DEGs were selected and further verified by RT-qPCR. Enrichment analyses of GO annotations and KEGG pathways revealed that these DEGs were associated with endocytosis, autophagy, cell adhesion, transport, immune response, apoptosis and so on. The expression of endocytosis-related genes, including CAV1/2, CAVIN2, Rab1B, CHMP2B/4C, VPS35, SNX2, Rab11B, CHMP6, MVB12B and VPS28, were found to be regulated. In addition, some genes associated with host immune defense, such as USP15, DHX29, DDX3, RIG-I and MDA5, were downregulated and the genes associated with host autophagy, such as WIPI2, ATG16L2, SMCR8, RPTOR and MLST8, were upregulated. Therefore, CSFV NS4B involved in virus invasion and intracellular trafficking, the induction of autophagy and the inhibition of antiviral response. Taken together, this study provides useful information for further understanding the function of NS4B during CSFV infection.

## 1 Introduction

Classical swine fever (CSF) is a contagious and fatal viral disease in pigs caused by the classical swine fever virus (CSFV), which leads to a huge economic loss in the pig industry (Dreier et al., 2007; Luo et al., 2014). Major symptoms of CSF include hyperthermia and systemic bleeding. Although large-scale outbreaks have been controlled in the past, elimination of CSF remains a challenge for many countries because of chronic and persistent infections (Luo et al., 2017). Therefore, further research of the pathogenic mechanism of CSFV is beneficial for the effective prevention and control of CSF.

CSFV is a single-stranded, positive-sense RNA virus and enveloped RNA virus, belonging to the *Pestivirus* genus within the *Flaviviridae* family (Ji et al., 2015). The genome of CSFV is 12.3 kb in length and contains a 5′-untranslated regions (UTR), a large open reading frame (ORF), and a 3′-UTR. The ORF encodes a polyprotein precursor, which is further proteolytically processed into four structural proteins (C, E^rns^, E1, and E2) and eight non-structural proteins (N^pro^, p7, NS2, NS3, NS4A, NS4B, NS5A, and NS5B) (Thiel et al., 1991; Lamp et al., 2011). CSFV non-structural proteins play an important role for viral replication, pathogenicity and regulation of host cell function.

CSFV NS4B is a membrane-associated multifunction viral protein, which exhibits NTPase activity and plays an important role for virus replication and infectious virion production (Moulin et al., 2007; Gladue et al., 2011; Ji et al., 2015). The C-terminal region of NS4B contains a TIR-like domain, and the mutation of TIR-like domain attenuates virulence and reduces virus growth (Fernandez-Sainz et al., 2010; Tamura et al., 2012). The N-terminal domain of CSFV NS4B determines viral genome replication and virulence (Tamura et al., 2015). Furthermore, interacting proteins of NS4B, such as Rab5, Rab22a, pRNF114, TBK1, RPLP1, show that NS4B is involved in viral replication, internalization and intracellular transportation (Lin et al., 2017; Lv et al., 2018a; Zhang et al., 2019; Dong et al., 2022; Wang et al., 2022; Zhang et al., 2022). However, the function of NS4B in other stages of the virus life cycle, such as autophagy and endocytosis, needs to be further understood.

High-throughput RNA sequencing (RNA-seq) enables investigation of any transcriptome at a fine resolution, which is used for accurate quantification of gene expression to identify the differentially expressed genes (DEGs) in different samples (He et al., 2017b; Park et al., 2021). The study aimed to investigate the functional role of CSFV NS4B in 3D4/21 cells by RNA-seq. This work provides novel information for further understanding NS4B-host interactions in virus life cycle.

## 2 Materials and methods

### 2.1 Cell culture and plasmid construction

3D4/21 cells (ATCC, CRL-2845) were cultured in RPMI 1640 medium (Gibco, UK) containing 10% fetal bovine serum (FBS) (Biowest, France) and 1% penicillin-streptomycin solution at 37°C and 5% CO_2_. pcDNA3.1-NS4B-Flag encoding the CSFV NS4B was constructed by cloning the NS4B gene into the plasmid vector pcDNA3.1(-) with a Flag-tag. The recombinant plasmid was confirmed by restriction digestion and sequencing.

### 2.2 The construction of 3D4/21 cells expressing NS4B

A total of 3 × 10^5^ cells/well were inoculated on the cell culture 6-well plate. After seed cells to be 70% confluent, 3D4/21 cells were transfected with the pcDNA3.1-NS4B-Flag plasmids (4 μg) using Lipofectamine 2000 (Invitrogen, USA), and pcDNA3.1(-) was used as a negative control. After 48 h, the cells were harvested. To confirm NS4B expression in 3D4/21 cells, NS4B gene expression was analyzed by RCR and Western blot. In brief, total cellular RNA was extracted using Trizol. The cDNA was synthesized using the PrimeScript RT reagent kit (Takara Bio, Japan). The expression of NS4B was verified by PCR with the specific primer in Supplementary Table S1. In addition, 3D4/21 cells expressing NS4B were harvested and lysed. Protein samples were separated by 12% SDS-PAGE and transferred onto PVDF membranes (Millipore, USA). The membranes were incubated with an anti-β-actin mouse mAb or anti-Flag mouse mAb (Proteintech, China) at 4°C overnight, and then were incubated with HRP-conjugated goat anti-mouse IgG (1:5000) (Bioss, China) for 2 h at room temperature. Then, the signal was detected using Western blot analysis system.

### 2.3 RNA isolation and library preparation

Total RNA was isolated using the Trizol Reagent (Invitrogen, USA). Then, RNA purity and concentration were examined using NanoDrop spectrophotometer (Thermo Scientific, USA) and Qubit 4.0. RNA integrity and quantity were measured using the Agilent 2100 system. Three micrograms of RNA were used as input material for the RNA sample preparations. Sequencing libraries were generated according to the following steps. Through combining with the ploy A tail of mRNA by A-T complementary pairing, mRNA was enriched by using magnetic beads with Oligo(dT), which will remove the rRNA. Fragmentation was carried out using divalent cations under elevated temperature in fragmentation buffer. First strand was synthesized by random hexamers, and the second cDNA strand was synthesized by adding buffer, dNTPs, RNase H and DNA polymerase I. Double stranded cDNA was repaired and A was added to the 3′ end. Hieff NGS^®^ DNA Selection Beads were used for purification and fragment selection. After purification and fragment selection, the products were amplified and enriched by PCR. Qubit was used for quantitative. The target region library of the duplex was denatured, cycled, and digested to yield a single-stranded circular DNA. Single-stranded circular DNA is amplified by a rolling circle amplification (RCA), known as DNA nano balls (DNB). After library construction, qubit was used for quantitative.

### 2.4 RNA sequencing and differentially expressed genes analysis

After library preparation, the sequencing library was sequenced on DNBSEQ-T7 with PE150 model. Samples are sequenced on the platform to get image files, which are transformed by the software of the sequencing platform, and 150 bp paired-end reads were generated, and the original data in FASTQ format (Raw Reads) is generated. The low quality reads were removed from the datasets to get high quality sequence (Clean Reads) by using fastp software (v0.21.0). The filtering principle was removing reads containing adaptors, removing the reads with more than 10% N ratio, and removing reads with more than 50% base of low quality (mass value < 20). Then, quality control of the clean data was tested by FastQC software. If qualified, the clean data was used for subsequent analysis. The clean reads were mapped to the Sus scrofa genome (GCF_000003025.6_Sscrofa11.1) using HISAT2 v2.1.0 (Kim et al., 2015). FPKM (Roberts et al., 2011) of each gene was calculated using Cufflinks (Trapnell et al., 2010), and the read counts of each gene were obtained by HTSeqcount (Anders et al., 2015). Differential expression analysis was analyzed by DESeq2 (v1.30.1) with screened conditions as follows: expression difference multiple |log_2_FoldChange| > 1, significant padj ≤ 0.05.

### 2.5 GO functional enrichment and pathway analysis

Hierarchical cluster analysis of DEGs was performed to demonstrate the expression pattern of genes in different groups and samples. All the genes were mapped to Terms in the Gene Ontology database (http://www.geneontology.org/) and calculated the numbers of differentially enriched genes in each term. Using cluster Profiler to perform GO enrichment analysis on the differential genes, calculate *P*-value by hypergeometric distribution method (the standard of significant enrichment is padj ≤ 0.05), and find the GO term with significantly enriched differential genes to determine the main biological functions performed by differential genes. ClusterProfiler (v3.18.1) software was used to carry out the enrichment analysis of the KEGG pathway of differential genes, focusing on the significant enrichment pathway with padj ≤ 0.05.

### 2.6 Real-time quantitative PCR (RT-qPCR) to validate gene expression

To validate the RNA-seq results, 12 genes were selected and verified by RT-qPCR with the specific primers in Supplementary Table S1. Total cellular RNA was extracted with Trizol (Invitrogen, USA). The cDNA was synthesized using the PrimeScript RT reagent kit (Takara Bio, Japan). The relative mRNA expression was calculated with an ABI7500 system (Biosystem, CA, USA), with β-actin as the reference, using UltraSYBR Mixture (CWBIO, China) according to the manufacturer's protocol. The data were analyzed by using the 2^−Δ*ΔCt*^ method. All experiments were performed in triplicate, and the results represent the mean ± standard deviation (SD).

### 2.7 Statistical analysis

The RT-qPCR experiments were performed at least three times, and the results represent the mean ± standard deviation (SD) of three replicates. The data were analyzed by one-way ANOVA and Bonferroni post hoc test using SPSS software (version 18.0). A *P* value < 0.05 was considered to indicate significance.

## 3 Results

### 3.1 Detection of NS4B expression in 3D4/21 cells

**Figure 1:**
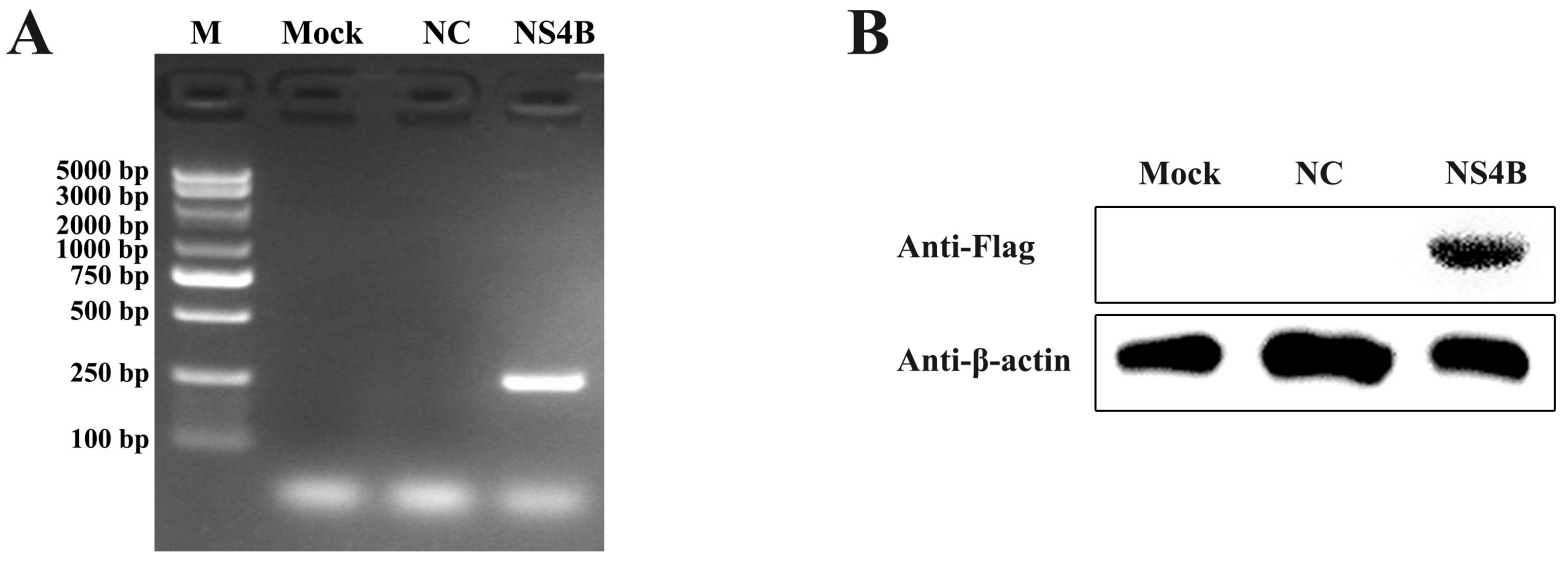
Detection of NS4B expression in 3D4/21 cells. **(A)** Validation of NS4B expression in 3D4/21 cells by PCR. Mock represents 3D4/21 cells; NC represents 3D4/21 cells transfected with empty vector; NS4B represents 3D4/21 cells transfected with pcDNA3.1-NS4B-Flag. **(B)** Validation of NS4B expression in 3D4/21 cells by Western blot. β-actin was used as an internal control.

3D4/21 cells were transfected with pcDNA3.1-NS4B-Flag for 48 h. Subsequently, the NS4B expression was detected by PCR and Western bolt. As shown in Figure 1A, the expression of the NS4B gene was detected by PCR. Additionally, NS4B expression was also confirmed by Western blot using anti-Flag mAb in NS4B-transfected cells (Figure 1B). These results indicated that CSFV NS4B was successfully expressed in 3D4/21 cells.

### 3.2 RNA sequencing and read assembly

In this study, sequencing libraries were prepared in triplicate. A total of 411,472,024 raw reads, an average of 68,578,671 reads per sample, were obtained from transcriptome sequencing from DNBSEQ-T7 platform, and were available for further expression level analysis after quality control. After discarding ribosomal RNA and low quality reads, an average of 68,578,605 readings per sample and an average of 10.20 gigabasepairs (Gbp) per sample were generated. Q30 percentages of clean reads for all samples were higher than 92.65%, and the GC contents of the clean reads for all samples ranged between 49.87% and 55.66% (Table 1). In addition, 94.29%−95.32% of clean reads of the sequences were mapped onto the reference genome Sus scrofa (GCF_000003025.6_Sscrofa11.1). Among the positioning reads, 82.08%−88.88% of clean reads were uniquely mapped to the reference genome Sus scrofa (GCF_000003025.6_Sscrofa11.1) (Table 1).

**Table 1:** Statistics of the RNA-seq datasets.

**Sample**	**Total reads**	**Clean reads**	**Clean bases (Gbp)**	**Total mapped reads**	**Uniquely mapped**	**Q30 rate**	**GC rate**
NC-1	78,001,888	78,001,806	11.59	94.56%	85.39%	93.68%	49.96%
NC-2	81,172,854	81,172,788	12.05	94.29%	82.08%	93.92%	52.41%
NC-3	72,474,214	72,474,146	10.80	95.06%	86.10%	92.65%	49.87%
NS4B-1	62,562,080	62,562,022	9.30	94.77%	88.27%	93.18%	53.09%
NS4B-2	62,206,422	62,206,374	9.26	95.32%	88.88%	93.21%	53.98%
NS4B-3	55,054,566	55,054,494	8.17	94.82%	87.54%	93.78%	55.66%

### 3.3 DEGs in NS4B-transfected 3D4/21 cells

**Figure 2:**
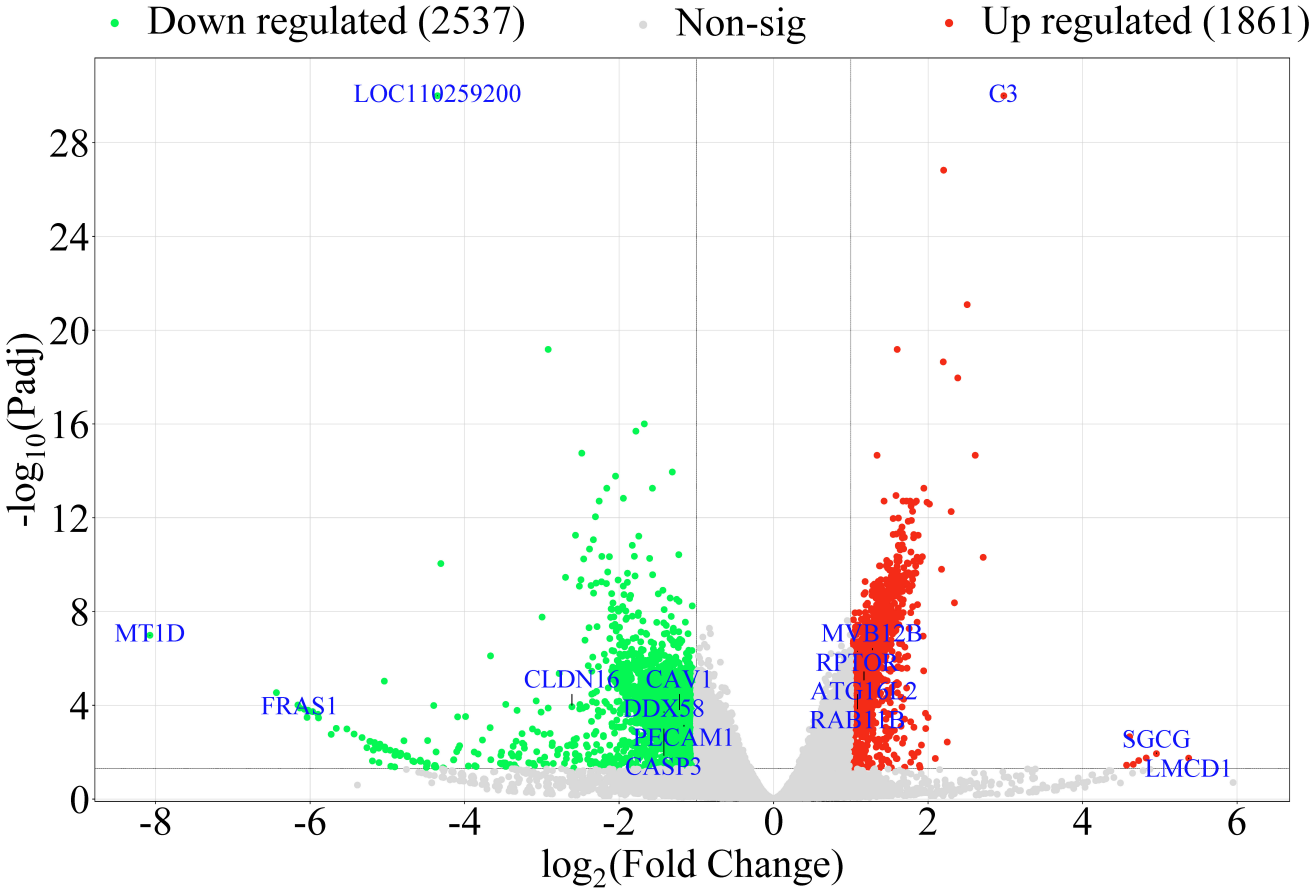
Volcano plot displaying the DEGs in NS4B-transfected 3D4/21 cells. In the figure, the two vertical black lines are the threshold of 2 times differential expression, and the horizontal black line is the threshold of *P-*adj = 0.05. Red dots indicate upregulated genes, green dots indicate downregulated genes, gray dots indicate non-significantly expressed differential genes. DEGs numbers are labeled on the top of the volcano plot.

To compare DEGs in NC with 3D4/21 cells transfected with NS4B, DEGs were performed in volcano plots. Differential expression analysis identified 4397 DEGs in NS4B-transfected 3D4/21 cells compared with NC cells, and 1861 DEGs were found to be upregulated and 2536 DEGs were downregulated (Figure 2). The horizontal axis was Log_2_(Fold Change), and the farther dots from the center represent greater difference multiple. The vertical axis is -Log_10_(padj), and the closer dots from the top of the graph represent the more significant difference. The significantly upregulated genes were C3, LMCD1, ATG16L2, RPTOR, RAB11B, MVB12B, SGCG, et al. The significantly downregulated genes were MT1D, CAV1, PECAM1, CLDN16, DDX58, CASP3, FRAS1, LOC110259200, et al. These DEGs were involved in endocytosis, autophagy, cell adhesion, transport, immune response, apoptosis and so on. Upregulated and downregulated DEGs are listed in Supplementary Table S2. The most upregulated gene in NS4B-transfected 3D4/21 cells was found to be LIM and cysteine-rich domains protein 1 (LMCD1), which increased 41.5-fold change. The most downregulated gene in NS4B-transfected 3D4/21 cells was found to be metallothionein-1D (MT1D), which reduced 269-fold change.

**Figure 3:**
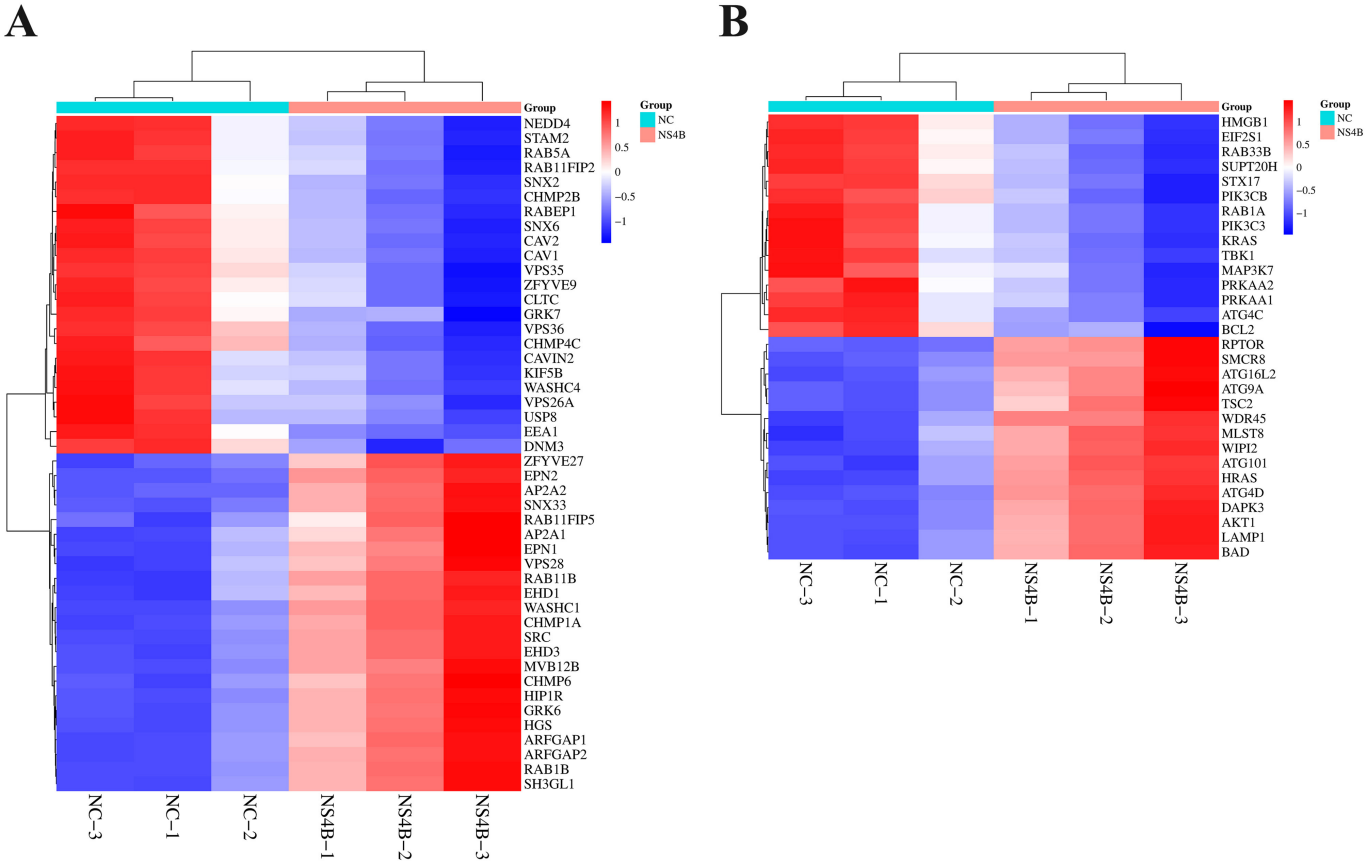
Expression profiles (heat maps) of endocytosis and autophagy genes in NS4B-transfected 3D4/21 cells. The samples NC-1, NC-2, NC-3 and NS4B-1, NS4B-2, NS4B-3 are similar but three independent experiments. Each column is a sample and represents the expression level of the same gene in different samples. The color in the heat map represents gene expression changes. Red indicates high gene expression, green indicates low gene expression and white indicates gene expression that has not been altered. DEGs are labeled on the right of the heat-map. **(A)** The heat maps of endocytosis genes. **(B)** The heat maps of cell autophagy genes.

Furthermore, 46 DEGs related to endocytosis and 30 DEGs related to autophagy were screened and further analyzed according to hierarchical clustering (Figures 3A, B). The important genes related to endocytosis were downregulated, such as CAV1/2, CAVIN2, Rab1B, CHMP2B/4C, VPS35, and SNX2. The upregulated important genes related to endocytosis were Rab11B, CHMP6, MVB12B, and VPS28. WIPI2, ATG16L2, SMCR8, RPTOR and MLST8, the important autophagy-related genes, were significantly upregulated in NS4B-transfected 3D4/21 cells. The analysis of RNA-seq data revealed that CSFV NS4B was involved in autophagy and endocytosis.

### 3.4 GO analyses and KEGG pathway analysis of DEGs

**Figure 4:**
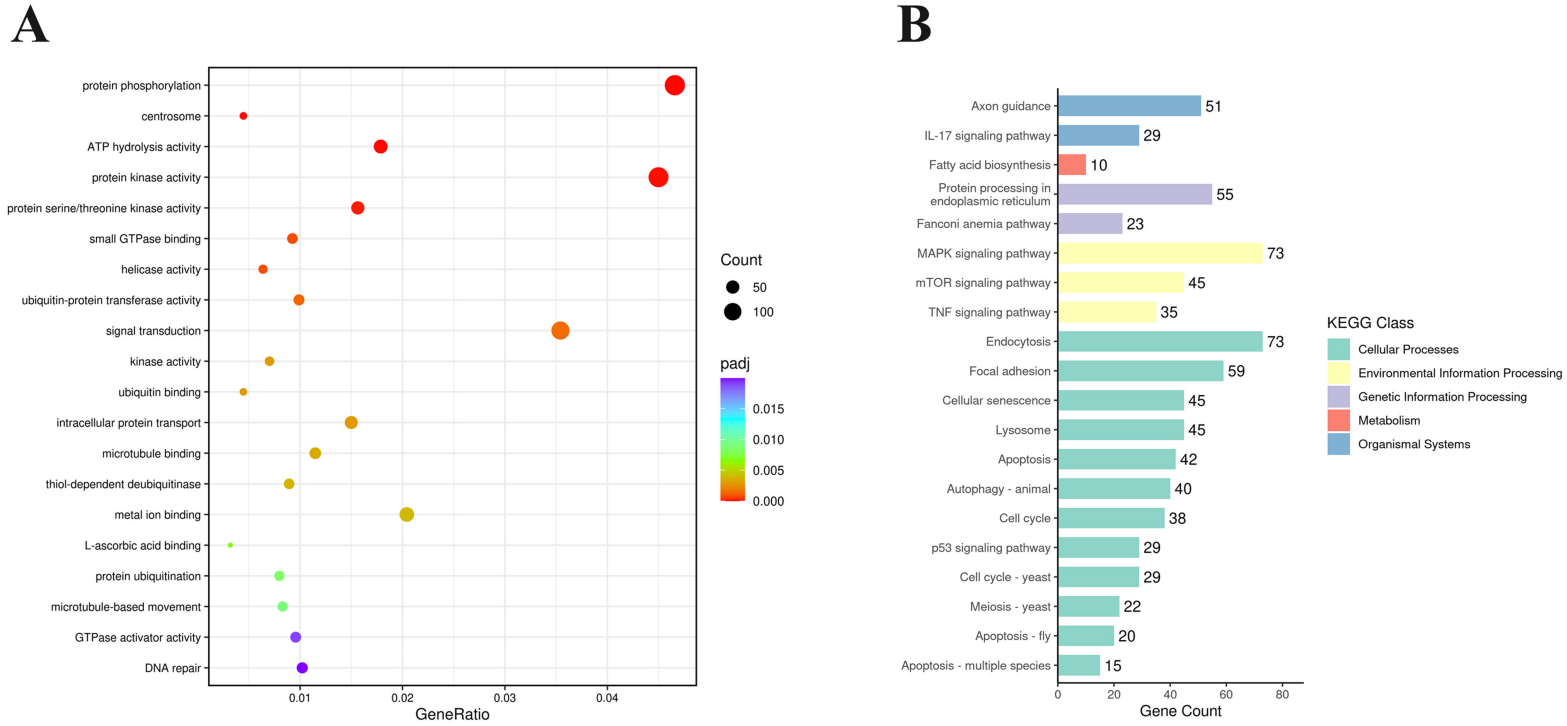
Functional enrichment analysis of DEGs expressed in NS4B-transfected 3D4/21 cells. **(A)** Statistics of GO term enrichment (Top 20). The top 20 GO terms with the smallest *P*-adj were selected. **(B)** Enriched KEGG pathways (Top 20). KEGG pathways were classified into 5 categories, including Cellular Processes, Environmental Information Processing, Genetic Information Processing, Metabolism, and Organismal Systems. The top 20 KEGG pathways with the smallest *P*-adj were selected.

To explore the biological functions of the DEGs, GO (Gene Ontology) enrichment analysis was conducted. These DEGs were classified into three main categories: biological process (BP), cellular component (CC), and molecular function (MF). Supplementary Table S3 showed that these DEGs were enriched in 468 GO terms, including 58 GO terms in CC category, 224 GO terms in MF category, and 186 terms in BP category. According to *P* < 0.05 as the standard, these DEGs were significantly enriched in 31 GO terms and the top 20 GO terms were listed in Figure 4A. The most significantly enriched term was protein phosphorylation (GO: 0006468), followed by protein kinase activity (GO: 0004672), signal transduction (GO: 0007165), and so on. Many DEGs are enriched in endocytosis (GO: 0006897), RNA helicase activity (GO: 0003724), regulation of apoptotic process (GO: 0042981) and autophagy (GO: 0006914), such as SNX2, DDX3X, DHX29, MDM2, CASP8, WIPI2, and so on.

Analysis of the KEGG pathway is used for predicting biological processes and phenotypic traits of genes. To analyze the function of CSFV NS4B, these DEGs were mapped to referential canonical signaling pathways via KEGG database analysis. According to *P* < 0.05 as the standard, DEGs were significantly enriched in 50 pathways and the top 20 enriched KEGG pathways were showed in Figure 4B. The top 20 enriched KEGG pathways contained endocytosis, MAPK signaling pathway, focal adhesion, protein processing in endoplasmic reticulum, cellular senescence, lysosome, mTOR signaling pathway, apoptosis, autophagy, TNF signaling pathway, p53 signaling pathway, etc. The most significant pathway was endocytosis (map04144), and 73 genes were differentially expressed. In addition, apoptosis (map04210) and autophagy-animal (map04140) were found in these pathways, which were related to apoptosis and autophagy research.

### 3.5 Validation of RNA-seq data by RT-qPCR

**Figure 5:**
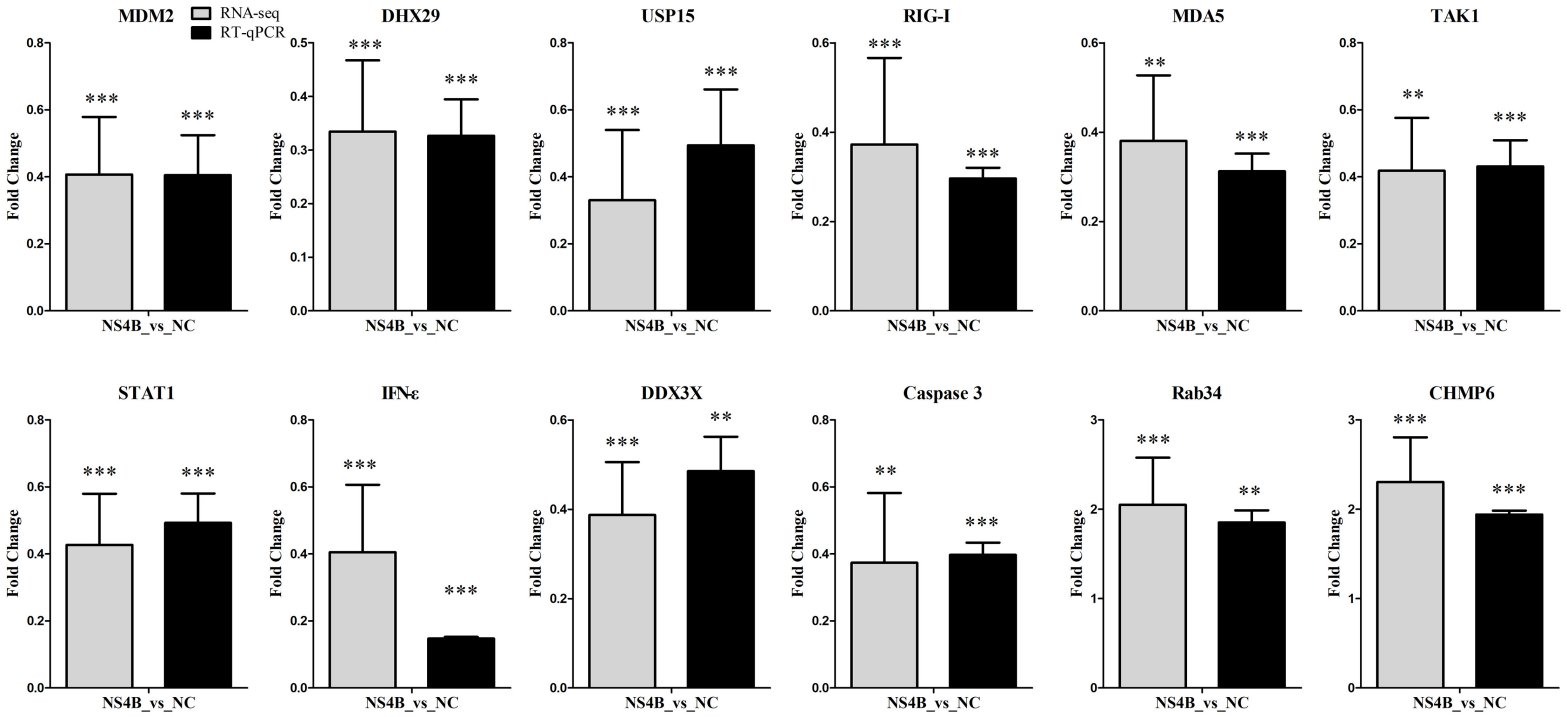
Validation of RNA-seq data by RT-qPCR. To confirm results from RNA-seq, RT-qPCR were performed using total RNAs from NS4B or NC expressing 3D4/21 cells. β-actin was used for reference. The y-axis represents normalized fold changes of these selected genes expression (NS4B vs. NC). The error bars represent the mean ± SD (*n* = 3). Significant discrepancies (“** or ***”) represents the comparison between NS4B and NC of RNA-seq or RT-qPCR results. **P < 0.01; ***P < 0.001.

To validate gene expression results obtained from RNA-seq, we tested the expression of important innate immune response genes (DHX29, DDX3X, RIG-I, MDA5, TAK1, STAT1, IFN-ε), apoptosis related genes (MDM2, Caspase3), endocytosis genes (Rab34, CHMP6) and other genes (USP15) using RT-qPCR. Comparison of RNA-seq results and RT-qPCR results of the selected genes showed that the two detection methods were largely consistent (Figure 5).

## 4 Discussion

CSF is an ancient intensive infectious disease, which has inflicted serious financial burden to the pork industry around the world (Postel et al., 2018). Previous transcriptomics have provided some understanding of the pathogenesis of CSFV infection, but the functions of CSFV proteins need to be further studied. CSFV NS4B is a non-structural protein, which is essential for viral virulence and genome replication. More in-depth studies on NS4B function will be of great importance in elucidating the mechanisms of CSFV infection. RNA-seq provides an effective means for the systematic study of the function of CSFV NS4B.

In the study, 3D4/21 cells were transfected with the pcDNA3.1-NS4B-Flag plasmids using Lipofectamine 2000. The expression level of NS4B was higher at 48 hpt than that at 24 hpt by RT-qPCR and Western blot (data not shown). Therefore, the cells were harvested at 48 hpt, and CSFV NS4B was successfully expressed in 3D4/21 cells (Figure 1). RNA-seq revealed 4397 DEGs in NS4B-transfected 3D4/21 cells (Figure 2). These DEGs were related to endocytosis, autophagy, cell adhesion, transport, immune response, apoptosis and other terms (Supplementary Table S3). Complement C3 (C3) was the upregulated gene with the most significant difference. C3 plays a central role in the activation of the complement system and causes histamine release from mast cells and basophilic leukocytes via increasing vascular permeability (Zarantonello et al., 2023), which suggestted NS4B might be involved in adaptive immune response of host. LOC110259200 was the downregulated gene with the most significant difference. However, the function of LOC110259200 is still not clear. Fraser syndrome protein 1 (FRAS1) involves in extracellular matrix organization, and is required for the regulation of epidermal-basement membrane adhesion responsible for proper organogenesis during embryonic development (McGregor et al., 2003). FRAS1 was significantly downregulated, which suggested that NS4B might involve in the lesion of embryo during CSFV infection. Sarcoglycan gamma (SGCG) is a subcomplex of the dystrophin-glycoprotein complex which forms a link between the F-actin cytoskeleton and the extracellular matrix (Vainzof et al., 2021). SGCG was significantly upregulated in NS4B-transfected 3D4/21 cells. MT1D were significantly downregulated, which has a high content of cysteine residues that bind various heavy metals. CSFV infection causes diffuse hemorrhaging in the skin, kidneys, and other organs of pigs (Moennig et al., 2003). LMCD1 mediates IL-33 expression, which plays a role in endothelial dysfunction (Govatati et al., 2019). LMCD1 were significantly upregulated in NS4B-transfected 3D4/21 cells, which may contribute to diffuse hemorrhaging caused by CSFV infection. Platelet endothelial cell adhesion molecule-1 (PECAM-1) was significantly downregulated in NS4B-transfected 3D4/21 cells, which plays an important role for maintaining and restoring the vascular permeability barrier (Lertkiatmongkol et al., 2016). The tight junction protein claudin (CLDN) plays a critical role in the regulation of paracellular permeability to ions and small molecules in endothelia or epithelia and the maintenance of cell polarity (Kwon, 2013), and CLDN12/16 were found to be downregulated in NS4B-transfected 3D4/21 cells (Supplementary Table S2). These molecules contribute to the maintenance and regulation of vascular permeability, suggest CSFV NS4B may be related to diffuse hemorrhaging of CSFV infection.

Compared to direct fusion at the plasma membrane, endocytosis is the preferred means of entry into the target cell (Kalia and Jameel, 2011). After attachment, CSFV internalizes via clathrin-mediated endocytosis (CME) and/or caveolae/raft-dependent endocytosis (CavME) (Guo et al., 2023). In the study, the analysis of RNA-seq data showed that many DEGs were involved in endocytosis (Figure 3A). CSFV particles are internalized via a caveolae-dependent pathway in 3D4/21 cells (Ning et al., 2016). Caveolae are mainly composed of lipids and proteins (caveolin, flotillin, etc.). Caveolins, including caveolin-1,−2, and−3 (CAV1, CAV2, and CAV3), are marker proteins of caveolae (Kurzchalia and Parton, 1999). CAV1 and CAV2 form a stable heterooligomeric complex that targeting to lipid rafts and drive caveolae formation and mediate the recruitment of caveolae associated proteins (CAVIN) to the caveolae (Bastiani et al., 2009). Analysis of the DEGs showed that CAV1/2 and CAVIN2 were downregulated in NS4B-transfected 3D4/21 cells. The result indicated that NS4B might inhibit CSFV invasion via suppressing caveolae-dependent pathway in the late stages of viral infection, which might help that the extracellular virion infected uninfected cells.

CSFV transports from early endosomes (EEs) to late endosomes (LEs) and LEs to the lysosomes along microfilaments with the assistance of ras-related proteins (Rabs) and endosomal sorting complex required for transport (ESCRT) proteins (Tsg, CHMP, etc.). In the ER lumens, vacuolar protein sorting-associated proteins (VPS) play an important role in the formation of CSFV replication complexes together with the ESCRT proteins. CSFV transports from ER to the Golgi and budded with the assistance of sorting nexin (SNX) and multivesicular bodies (MVBs). The small GTPases Rab are key regulators of intracellular membrane trafficking. Rab5, Rab7, and Rab11 guide CSFV virions through the transport route from early to late and even recycling endosomes, followed by a transfer to lysosomes before viral RNA release (Guo et al., 2023). Charged multivesicular body protein 2B/4C/6 (CHMP2B/4C/6) are peripherally associated component of the endosomal sorting required for transport complex III (ESCRT-III) which is involved in MVBs formation and sorting of endosomal cargo proteins into MVBs. VPS35, a retromer subunit, is recruited to viral replication sites to promote HCV replication (Yin et al., 2016). VPS28 plays an important role in the formation of CSFV replication complexes together with CHMP2B/4B (Liu et al., 2022). MVB12A and MVB12B constitute ESCRT-I subunits and play a unique role in regulating ESCRT-mediated HIV budding (Morita et al., 2007). SNX proteins involve in cargo recognition and sorting during retrograde transport from the endosome to the Golgi complex (Kocmar et al., 2022). RNA-seq data showed that several key genes were downregulated, including Rab1B, CHMP2B/4C, VPS35 and SNX2. In addition, Rab11B, CHMP6, MVB12B, and VPS28 were upregulated. These findings indicated that CSFV NS4B involved in intracellular trafficking by regulating the expression of Rabs and ESCRT genes, which suggested that NS4B might promote virus replication by regulating intracellular trafficking of CSFV particles.

Autophagy is an intracellular recycling and degradation pathway and plays important roles in cell survival and maintenance (Parzych and Klionsky, 2014). CSFV infection triggers a complete autophagic response to promote virus replication (Pei et al., 2014). RNA-seq results showed that the expression of some key autophagy-associated genes, such as WD repeat domain, phosphoinositide interacting 2 (WIPI2), autophagy related 16 like 2 (ATG16L2), Smith-Magenis syndrome chromosomal region candidate gene 8 (SMCR8), regulatory associated protein of MTOR complex 1 (RPTOR) and MLST8, were increased in NS4B-expressing cells (Figure 3B). WIPI2b directly binds Atg16L1, and results in LC3 conjugation and starvation-induced autophagy (Dooley et al., 2014). SMCR8 has distinct roles in different phases of autophagy including autophagosome formation and maturation (Jung and Behrends, 2020). RPTOR and MLST8 induce autophagy by phosphorylating the autophagy initiation components ULK1 and ATG13 (Coffman et al., 2014). These results indicated that NS4B contributed to the induction of autophagy, which might subsequently promote virus replication during CSFV infection.

GO enrichment analysis showed that 146 DEGs were significantly enriched in protein phosphorylation (GO: 0006468) and 141 DEGs were significantly enriched in protein kinase activity (GO: 0004672), such as MAP3K11, AKT2, JAK2, and so on (Figure 4A). Mitogen activated protein kinase 11 (MAP3K11) is a member of the serine/threonine kinase family and is required for serum-stimulated cell proliferation and for mitogen and cytokine activation of MAPK14 (p38), MAPK3 (ERK) and MAPK8 (JNK1) through phosphorylation and activation of MAP2K4/MKK4 and MAP2K7/MKK7 (Chadee and Kyriakis, 2004). RAC-beta serine/threonine-protein kinase (AKT2) is a serine/threonine kinase closely related to AKT1 and AKT3, known as AKT kinase, and regulates many processes, including metabolism, proliferation, cell survival, growth and angiogenesis, through the phosphorylation of a range of downstream substrates (Heron-Milhavet et al., 2011). Janus kinase 2 (JAK-2) is a non-receptor tyrosine kinase involved in various processes such as cell growth, development, differentiation or histone modifications and mediates essential signaling events in both innate and adaptive immunity (Royer et al., 2005). In addition, 111 DEGs were significantly enriched in signal transduction (GO: 0007165), such as TRAF2, FADD, SNX17, STAT1, TLR3, MAP3K7, etc. These genes are involved in Toll-like receptors (TLRs), apoptosis, endosomal recycling, defense response to virus, mitogen activated protein kinase (MAPK) signal transduction, and so on (Ninomiya-Tsuji et al., 1999; Stockinger et al., 2002). These findings indicated NS4B might regulate some DEGs having protein kinase activity to catalyze intracellular protein phosphorylation and activate some signaling transduction, which might change cellular physiological processes and contribute to the replication and pathogenesis of CSFV. Furthermore, 15 DEGs were enriched RNA helicase activity (GO: 0003724), such as DDX3X, DHX29, etc. DExH-Box helicase 29 (DHX29), an RNA co-sensor, specifically interacts with MDA5 to enhance antiviral immunity (Zhu et al., 2018). DEAD/H BOX 3 (DDX3, DDX3X) helicase binds the MAVS to upregulate IFN-β-inducing antiviral immunity (Oshiumi et al., 2010). These findings indicated NS4B might involve in CSFV replication via regulating these DEGs having RNA helicase activity.

Analysis of the KEGG pathway showed that 73 DEGs were significantly enriched in MAPK signaling pathway (Figure 4B). MAPK is a major cell signaling pathway that is known to mediate a variety of biological processes, such as inflammation, cell proliferation, apoptosis and differentiation, aging and tumorigenesis. The activation of MAPK is a key event for viral replication (Cheng et al., 2020). The inhibition of p38 MAPK activation suppressed CSFV replication (Lv et al., 2018b). Fifty-nine DEGs were significantly enriched in focal adhesion. Focal adhesion plays a major role in cell migration, cell cycle progression, proliferation, differentiation, growth and repair (Mishra and Manavathi, 2021). Fifty-five DEGs were significantly enriched in protein processing in endoplasmic reticulum (ER). Eukaryotic cells possess specialized machineries to ensure that the ER enables the proteins to acquire adequate folding and maturation for maintaining protein homeostasis. However, a large variety of physiological and pathological perturbations lead to the accumulation of misfolded proteins in the ER, which is referred to as ER stress (Moon et al., 2018). The ER stress induced by CSFV can promote CSFV production (He et al., 2017a). These findings indicated that NS4B affected a variety of intracellular biological processes by MAPK signaling pathway, focal adhesion and protein processing in endoplasmic reticulum and might contribute to the replication and pathogenesis of CSFV. In addition, many DEGs were significantly enriched in cellular senescence, lysosome, mTOR signaling pathway, TNF signaling pathway and p53 signaling pathway, which might contribute to the pathogenesis of CSFV.

During viral infection, viral RNA can be recognized by cytoplasmic cellular pattern-recognition receptors, such as the retinoic acid-inducible gene 1 protein (RIG-I, DDX58) and melanoma differentiation-associated protein 5 (MDA5), and activate mitochondrial antiviral signaling protein (MAVS) to induce antiviral and pro-inflammatory factors. The ubiquitin-specific protease 15 (USP15) promotes RIG-I-mediated antiviral signaling (Pauli et al., 2014). However, CSFV has strategies to evade the host antiviral immune system during a natural infection and establishes a persistent infection (Johns et al., 2010; Dong et al., 2018). In the study, USP15, DHX29, DDX3, RIG-I and MDA5 expression were downregulated in NS4B-transfected 3D4/21 cells (Figure 5). In addition, antiviral genes transforming growth factor-beta-activated kinase 1 (TAK1), interferon epsilon (IFN-ε) and signal transducer and activator of transcription 1 (STAT1) were also downregulated. TAK1 plays a signaling hub in innate immune and pro-inflammatory signaling pathways. IFN-ε directly mediates protection against viral infections (Tasker et al., 2016). STAT1 binds to the IFN stimulated response element (ISRE) to activate the transcription of IFN-stimulated genes (ISG), which drive the cell in an antiviral state (Greenlund et al., 1994). The results suggested that CSFV NS4B might contribute to immune escape of CSFV by reducing the expression of these antiviral genes. *In vitro*, CSFV infection does not induce apoptosis, and the effect of CSFV NS4B on apoptosis is unknown. In the study, MDM2 and Caspase3 expression were downregulated in NS4B-transfected 3D4/21 cells. The MDM2 binds NDUFS1 and induce apoptosis (Elkholi et al., 2019). Caspase3, a cysteine-aspartic acid protease, plays a central role in the execution-phase of cell apoptosis. The results suggested that NS4B might involve in the inhibition of cell apoptosis during CSFV infection.

In summary, this study revealed 4397 DEGs in NS4B-transfected 3D4/21 cells via RNA-seq. These DEGs were involved in endocytosis, autophagy, cell adhesion, transport, immune response, apoptosis and other processes. The upregulation and downregulation of some DEGs indicated CSFV NS4B involved in the regulation of vascular permeability, virus invasion and intracellular trafficking, the induction of autophagy, the inhibition of antiviral response and cell apoptosis, which might contribute to virus proliferation and diffuse hemorrhaging during CSFV infection.

## Data Availability

The original contributions presented in the study are included in the article/Supplementary material, further inquiries can be directed to the corresponding authors.

## Supplementary material

Supplementary Table S1 Primers used in this study.

Supplementary Table S2 Upregulated and downregulated genes in CSFV NS4B expressing 3D4/21 cells compared to those in NC expressing 3D4/21 cells.

Supplementary Table S3 The GO terms of DEGs.

Supplementary Figure S1 The original blot image of NS4B-Flag in Figure 1B.

Supplementary Figure S2 The original blot image of β-actin in Figure 1B.
