# Supplementary material for: Transcriptome analysis of 3D4/21 cells expressing CSFV NS4B
Source: Front Microbiol. 2025 Feb 4;16:1510058. doi: 10.3389/fmicb.2025.1510058 (PMC11833225; doi:10.3389/fmicb.2025.1510058)
Supplement: Supplementary Table S1 — Primers used in this study. [file Table_1.docx]

**Supplementary Table 1** Primers used in this study

| **primers** | **Sequence (5′ → 3′)** | **Purpose** |
| --- | --- | --- |
| **NS4B-Flag-F** | GGAATTCATGGCTCAGGGGGATGTG | Amplification of NS4B |
| **NS4B-Flag-R** | CCGGATCCTTA*CTTATCGTCGTCATCCTTGTAATC*TAGCTGGCGGATCTTC |  |
| **NS4B-F** | GCTCAGGGGGATGTG | PCR for detection of NS4B |
| **NS4B-R** | AAGCCTGGCACTGATGCTCT |  |
| **MDM2-F** | AATGAATCCTCCCCTTCCAC | RT-qPCR for detection of MDM2 |
| **MDM2-R** | AGGGACATCAAAGCCCTCTT |  |
| **DHX29-F** | CACTGGCTATGGCAGACTCA | RT-qPCR for detection of DHX29 |
| **DHX29-R** | TCCTGCTGCCTTAACCAACT |  |
| **USP15-F** | TTACCTGACACTTCCATTGCCC | RT-qPCR for detection of USP15 |
| **USP15-R** | CTCCTGAAAGAGTAGACAACGC |  |
| **RIG-I-F** | CCTCGGTGGCAGATGAAG | RT-qPCR for detection of RIG-I |
| **RIG-I-R** | TCAGCGTTAGCAGTCAGAAG |  |
| **MDA5-F** | CTGCAGACGAAGTTTGCTGAC | RT-qPCR for detection of MDA5 |
| **MDA5-R** | TAGCTGGTGATGGGGTCCTC |  |
| **TAK1-F** | AGGTTGTTGGAAGAGGAGCC | RT-qPCR for detection of TAK1 |
| **TAK1-R** | GCCCCCTTCAGCATATTCCA |  |
| **STAT1-F** | AGCTTCAGCAGCTTGACTCC | RT-qPCR for detection of STAT1 |
| **STAT1-R** | AAAACGGATGGTGGCAAAC |  |
| **IFN-ε-F** | CTTCAGCCTCTTCAGGGCAGTT | RT-qPCR for detection of IFN-ε |
| **IFN-ε-R** | GACAATGGTCCAGGCACAGC |  |
| **DDX3X-F** | ACAGCAGTTTTGGATCCCGT | RT-qPCR for detection of DDX3X |
| **DDX3X-R** | ACGGTCATCAAACCTTCCCC |  |
| **Caspase3-F** | GGATTGAGACGGACAGTGGG | RT-qPCR for detection of Caspase3 |
| **Caspase3-R** | CCGTCCTTTGAATTTCGCCA |  |
| **Rab34-F** | TACGGGTGAAAATGTCCGGG | RT-qPCR for detection of Rab34 |
| **Rab34-R** | CACTGTTGAGACGGACAACG |  |
| **CHMP6-F** | ATAGAGGAGGTGGAGCGCAT | RT-qPCR for detection of CHMP6 |
| **CHMP6-R** | TGGGATCGTCTCAGGAAGGG |  |
| **β-actin-F** | CAAGGACCTCTACGCCAACAC | RT-qPCR for detection of β-actin |
| **β-actin-R** | TGGAGGCGCGATGATCTT |  |

Underlines show restriction enzyme sites, italic show flag tag.
