# Supplementary figures and images for: Transcriptome analysis of 3D4/21 cells expressing CSFV NS4B
Source: Front Microbiol. 2025 Feb 4;16:1510058. doi: 10.3389/fmicb.2025.1510058 (PMC11833225; doi:10.3389/fmicb.2025.1510058)

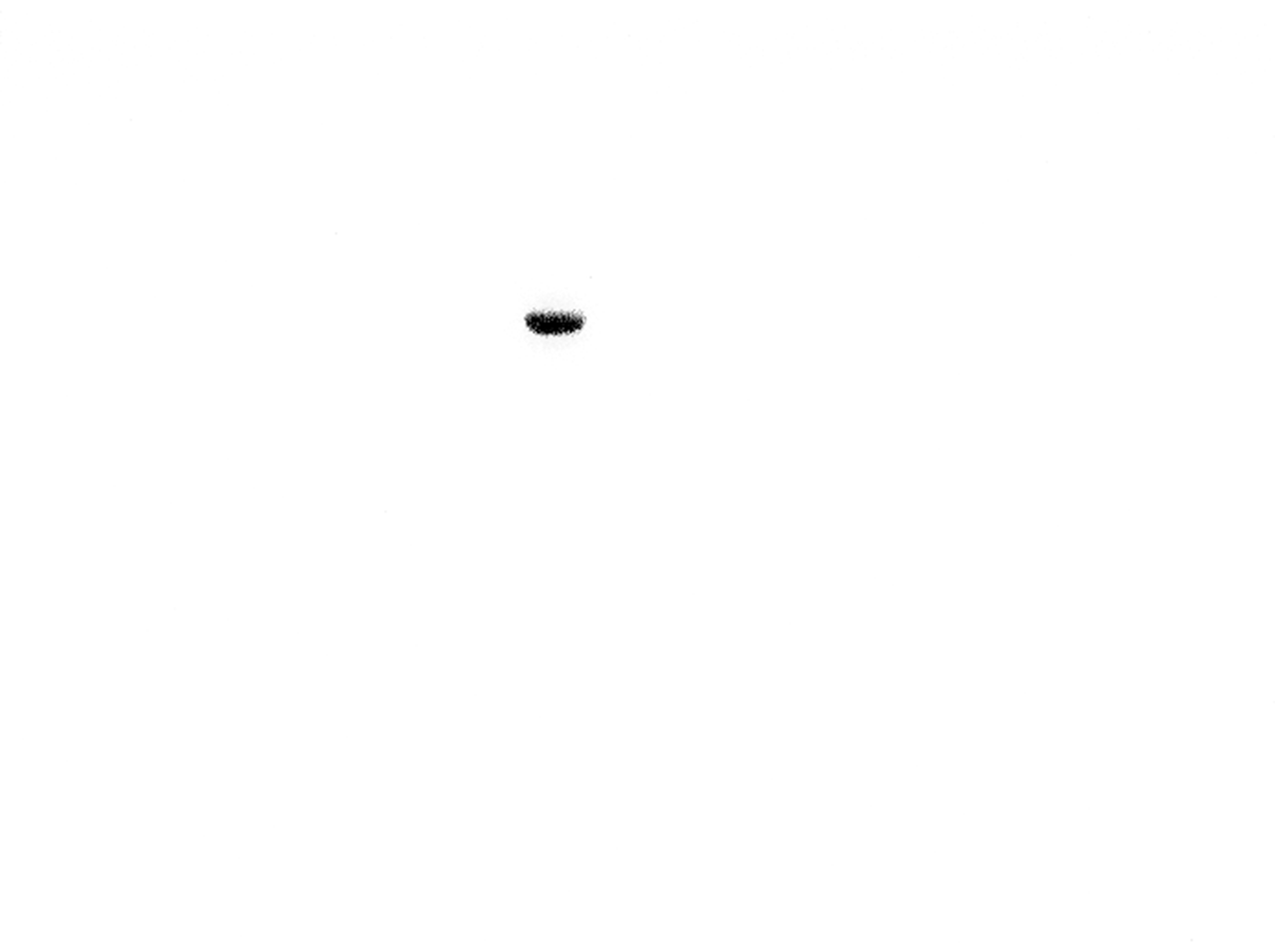

Supplement: Supplementary Figure S1 — The original blot image of NS4B-Flag in Figure 1B. [file Image_1.tif]

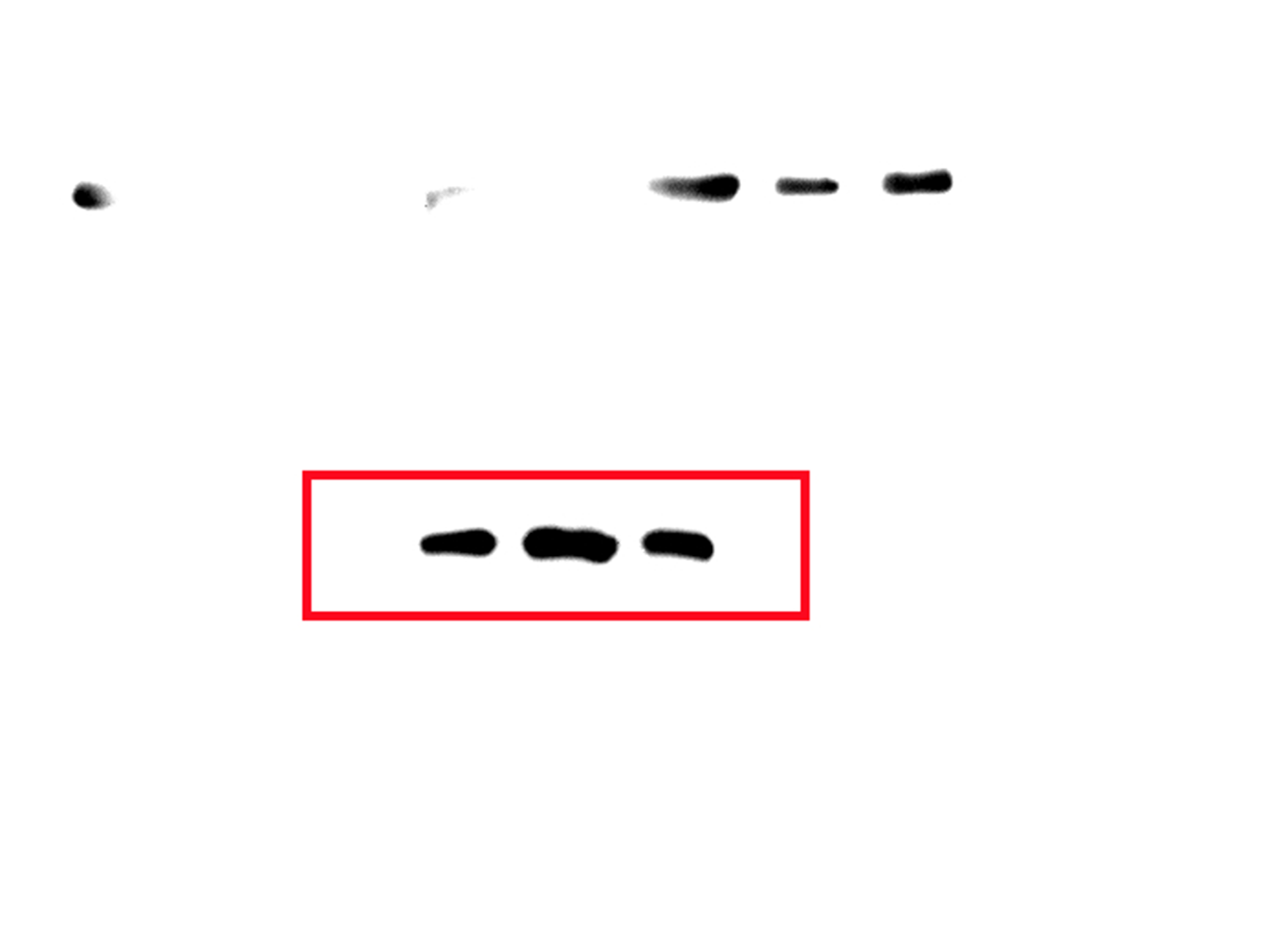

Supplement: Supplementary Figure S2 — The original blot image of β-actin in Figure 1B. [file Image_2.tif]
